# Silaphenolates and Silaphenylthiolates: Two Unexplored Unsaturated Silicon Compound Classes Influenced by Aromaticity

**DOI:** 10.3390/molecules17010369

**Published:** 2012-01-02

**Authors:** Alvi Muhammad Rouf, Henrik Ottosson

**Affiliations:** Department of Biochemistry and Organic Chemistry, Uppsala University, Box 576, 751 23 Uppsala, Sweden; Email: alvi.muhammadrouf@biorg.uu.se

**Keywords:** silicon, aromaticity, quantum chemical calculations

## Abstract

Monosilicon analogs of phenolates and phenylthiolates are studied by quantum chemical calculations. Three different silaphenolates and three different silaphenylthiolates are possible; the *ortho*-, *meta*-, and *para*-isomers. For the silaphenolates, the *meta*-isomer is the thermodynamically most stable, regardless if the substituent R at Si is H, *t*-Bu or SiMe_3_. However, with R = H and SiMe_3_ the energy differences between the three isomers are small, whereas with R = *t*-Bu the *meta*-isomer is ~5 kcal/mol more stable than the *ortho*-isomer. For the silaphenylthiolates the *ortho*-isomer is of lowest energy, although with R = H the *ortho*- and *meta*-isomers are isoenergetic. The calculated nucleus independent chemical shifts (NICS) indicate that the silaphenolates and silaphenylthiolates are influenced by aromaticity, but they are less aromatic than the parent silabenzene. The geometries and charge distributions suggest that all silaphenolates and silaphenylthiolates to substantial degrees are described by resonance structures with an exocyclic C=O double bond and a silapentadienyl anionic segment. Indeed, they resemble the all-carbon phenolate and phenylthiolate. Silaphenylthiolates are less bond alternate and have slightly more negative NICS values than analogous silaphenolates, suggesting that this compound class is a bit more aromatic. Dimerization of the silaphenolates and silaphenylthiolates is hampered due to intramolecular Coulomb repulsion in the dimers, and silaphenolates with a moderately bulky SiMe_3_ group as substituent at Si should prefer the monomeric form.

## 1. Introduction

Aromatic compounds with one or several silicon atoms in the aromatic ring have been investigated extensively during the last decades, and have recently been reviewed [1,2,3]. These silaaromatic compounds have a high aptitude for dimerization and very bulky substituents are needed to hinder this process [4]. The intriguing silaaromatic compounds of Tokitoh and co-workers, with just one large 2,4,6-*tris*[*bis*(trimethylsilyl)methyl]phenyl (Tbt) substituent, are especially notable [2,4,5,6].

**Scheme 1:**
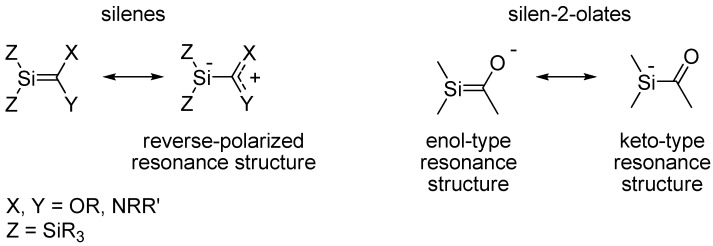
Resonance structures of silenes and silenolates.

In the context of unsaturated silicon compounds it is noteworthy that compounds with isolated Si=C double bonds, the so-called silenes [7,8,9,10,11,12], have a reduced tendency for dimerization when the Si=C bond is influenced by reverse Si=C bond polarity, *i.e.*, an Si^δ−^=C^δ+^ bond polarity rather than the natural Si^δ+^=C^δ−^ bond polarity [13,14]. Influence of reverse Si=C bond polarization can be induced by π-electron donating substituents at the C terminus and/or by σ-electron donating substituents at the Si terminus (Scheme 1) [13,15]. It has also been found that reverse polarization leads to a lower reaction rate for the addition of water and alcohols to silenes [7,16,17,18]. With regard to substituted silabenzenes we earlier found through quantum chemical calculations that reverse Si=C bond polarization could reduce their tendency for dimerization [19].

Some years ago we formed potassium 1,1-bis(trimethylsilyl)-2-*tert*-butylsilen-2-olate [20], a species which according to X-ray crystallography and computational studies is best described as an acyl substituted silyl anion with the negative charge at Si (Scheme 1). Moreover, this silenolate was stable at ambient temperatures for periods of months. Through quantum chemical computations we also noted significant differences between potassium and lithium silenolates [21], the latter being a compound class which earlier was examined independently by Apeloig, Bravo-Zhivotovskii and co-workers, and by Ishikawa, Ohshita and co-workers [22,23]. The recent X-ray crystal structures of two lithium silenolates by the group of Apeloig and Bravo-Zhivotovskii confirm the large structural differences between lithium and potassium silenolates [24]. In short, the lithium silenolates have a large influence of the enol-type resonance structure whereas potassium silenolates are dominated by the keto-type resonance structure (Scheme 1).

**Scheme 2:**
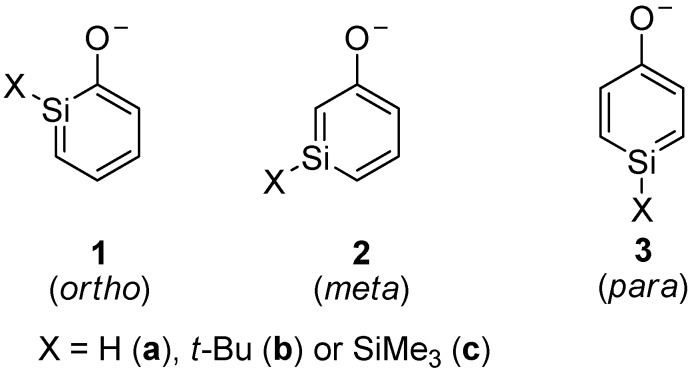
Isomers of silaphenolates.

**Scheme 3:**
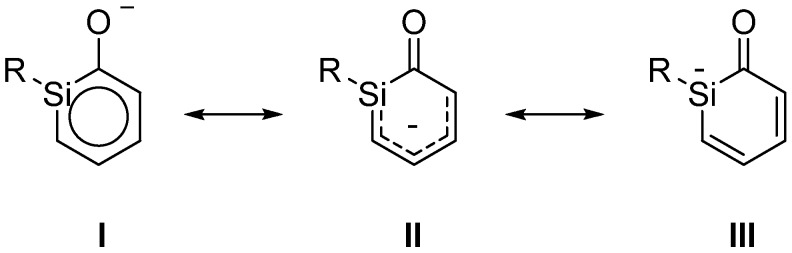
Resonance structures of *ortho*-silaphenolate.

Silaphenolates, the focus of the study reported herein, can be viewed as combinations of a silabenzene and a silenolate, and they constitute a class of compounds which hitherto has not been explored (Scheme 2). They should be categorized as unsaturated silicon compounds tentatively stabilized by aromaticity in case the negative charge primarily resides at the oxygen atom (resonance structure **I**, Scheme 3). However, the negative charge may also primarily be delocalized in the silapentadienyl moiety of the ring (resonance structure **II**). Thirdly, the *ortho*- and *para*-silaphenolates may be influenced by reverse polarization if the negative charge is extensively localized onto the silicon atom (resonance structure **III**). Regardless of how the negative charge is distributed, their dimerization should be hampered because of Coulomb repulsion in the dianionic dimer. Less bulky substituents may thus be required for these species to be isolable as monomers when compared to silabenzenes and other neutral silaaromatic compounds.

We have now explored silaphenolates as well as silaphenylthiolates through quantum chemical calculations. The calculations were carried out with four different methods so as to obtain a span in the method variations, thus allowing for conclusions that are made on computationally broader grounds. The properties examined include relative isomer energies, atomic charges, geometries, nucleus independent chemical shifts (NICSs), as well as dimerization energies. A question is to what extent the silaphenolates and silaphenylthiolates can be described as aromatic species? Moreover, will their dimerizations be hampered when compared to silabenzenes? In the *ortho*- and *para*-silaphenolates and -silaphenylthiolates, respectively, the negative charge can be localized to the silicon atom of the ring, thus leading to a decrease in the partial positive charge at this atom normally found for a Si atom in a neutral silaaromatic compound. This reverse polarization effect is not possible in the corresponding *meta*-isomers. However, will such reverse polarization effects be influential?

In addition to a study of the properties of the different isomers, we have also examined how substitution at Si changes the character of the various silaphenolates and silaphenylthiolates when compared to the model species with R = H. In this regard we have selected trimethylsilyl and *tert*-butyl groups as realistic and moderately bulky substituents. The hope is that our computational results can provide guidelines for later synthetic efforts as we explore which of the species studied herein have the highest possibilities to represent stable, monomeric and aromatic silaphenolates and silaphenyl-thiolates.

## 2. Computational Methods

Geometry optimizations of the silaphenolate and silaphenylthiolate monomers were first performed at the M062X/6-311G(d) hybrid meta density functional theory level [25,26,27]. Frequency calculations were also carried out at this level of computation in order to ensure that the structures discussed herein correspond to minima on the potential energy surface. Single-point energy calculations were subsequently made at M062X/6-311+G(d) level.

Additional geometry optimizations were performed at B3LYP/6-311G(d) [28,29], MP2/6-311G(d) and CCSD/6-311G(d) levels in order to examine the method variation in the calculated geometries. Results from the CCSD computations serve as benchmarks for the three inexpensive methods.

Atomic charges were calculated through natural population analysis (NPA) at all four levels of computation. NMR chemical shift calculations were performed with the gauge including atomic orbitals (GIAO) method at GIAO/M062X/6-311+G(d)//M062X/6-311G(d) level [30].

We also examined a few of the silaphenolate dimers, both without and with complexation by potassium ions solvated by THF molecules. These calculations were mainly performed at M062X/6-31G(d) level as M062X previously has been shown to be a good method to deal with nonbonded dispersive interactions [26]. However, in order to allow for comparisons with earlier computed data of silabenzene dimers we also carried out calculations of the silaphenolate dimers at B3LYP/6-31G(d) level.

All calculations were done with the Gaussian09 program package [31].

## 3. Results and Discussion

We first present and discuss the properties of silaphenolates and thereafter those of silaphenylthiolates. For each compound class we discuss the properties in the following order: relative isomer energies, geometries, charge distributions, NICS values, and dimerization energies.

### 3.1. Relative Energies of Silaphenolates

The energies of the *ortho*-isomers are taken as reference level for the relative energies. The calculations of the three isomers of the unsubstituted silaphenolates (**1a**, **2a** and **3a**) indicate that the M062X method is the inexpensive method which provides the best agreement in energies with the CCSD method (Table 1). For the three unsubstituted silaphenolates the *meta*-isomer **2a** is of lowest relative energy, whereas the *para*-isomer **3a** is of highest. The energy difference between the *ortho*- and *para*-isomers is, however, smaller than between the *ortho*- and *meta*-isomers.

Among the silaphenolates with substituents at Si, the *meta*-isomers are again the most stable isomers and the *para*-isomers are the least (Table 1). However, with R = SiMe_3_ (**1c**–**3c**) the three isomers are nearly isoenergetic, whereas with R = *t*-Bu (**1b**-**3b**) the energy difference between the *ortho*- and *meta*-isomers at B3LYP and MP2 (but not M062X) levels is smaller than between the *ortho*- and *para*-isomers, *i.e.*, opposite to the case when R = H. In conclusion, it is clear that the substituent at Si has an effect on the relative thermodynamic stabilities of various silaphenolates.

**Table 1:** Calculated relative energies of silaphenolates **1a**–**3c**^a^.

Compound	*E*_rel_				*H* ^298^	D*G*^298^
	M062X	B3LYP	MP2	CCSD	M062X	M062X
**1a**	0.0, *0.0*	0.0	0.0	0.0	0.0	0.0
**1b**	0.0, *0.0*	0.0	0.0	-	0.0	0.0
**1c**	0.0, *0.0*	0.0	0.0	-	0.0	0.0
**2a**	−5.8, *−5.8*	−4.1	−4.4	−7.0	−5.7	−5.2
**2b**	−4.3, *−5.0*	−2.8	−1.3	-	−4.7	−2.1
**2c**	−1.1, *−2.2*	0.0	0.4	-	−0.7	−3.2
**3a**	1.1, *1.4*	1.8	1.9	0.9	0.8	2.1
**3b**	5.5, *4.8*	5.3	5.8	-	4.6	6.2
**3c**	0.6, *0.5*	0.7	1.8	-	0.6	−0.1

^a^ Values in normal print obtained from geometry optimizations at the corresponding levels using the 6-311G(d) basis set, and values in italics obtained from single-point energy calculations using the 6-311+G(d) basis set.

### 3.2. Geometries of Silaphenolates

A comparison of the bond length data of the parent silaphenolates **1a**–**3a** obtained by the three inexpensive methods with those of CCSD reveal that B3LYP performs best overall [mean absolute deviations (MADs) of 0.006 (M062X), 0.004 (B3LYP), and 0.006 Å (MP2), respectively]. However, the three methods perform variously well for the three silaphenolates. MP2 has the smallest MAD for the *ortho*-silaphenolate (0.002 Å) but the largest for the *para*-silaphenolate (0.009 Å). With M062X the MADs for the three parent silaphenolate isomers are constant at 0.006–0.007 Å, while with B3LYP they are 0.004, 0.005, and 0.003 Å for **1a**, **2a**, and **3a**, respectively. The finding on the slightly better quality of the B3LYP geometries versus the M062X geometries agrees with an earlier finding reported by Zhao and Truhlar [26].

**Figure 1:**
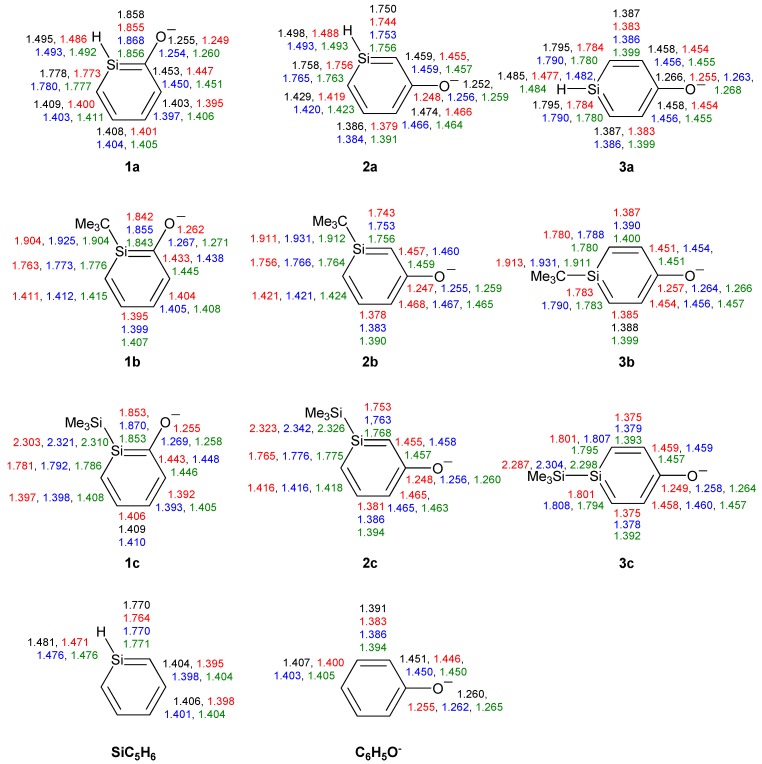
Bond lengths (Å) of silaphenolates **1a**–**3c**, silabenzene **SiC_5_H_6_** and all-carbon phenolate **C_6_H_5_O^−^** calculated at the CCSD/6-311G(d) (black), M062X/6-311G(d) (red), B3LYP/6-311G(d) (blue), and MP2/6-311G(d) (green) levels. All sums of valence angles at Si (Σα(Si)) equal 360.0°.

The SiC bond lengths of the three silaphenolates should be compared to those of the parent silabenzene (1.764–1.771 Å, Figure 1). Clearly, there are distinct differences in the SiC bond lengths of **1a**, **2a** and **3a**. The *ortho*-isomer **1a** has the longest SiC bond (the SiC(O) bond is nearly an Si–C single bond), and this bond would (tentatively) be the most markedly influenced by reverse polarization. As can be expected, the *meta*-silaphenolate **2a** reveals no influence of reverse polarizartion, and both SiC bonds are short (nearly Si=C double bonds). The *para*-isomer **3a** has SiC bond lengths which are only modestly elongated when compared to those of the parent silabenzene. Here, it should be noted that all three silaphenolates are planar at M062X, B3LYP, and MP2 levels (no frequency calculations performed at CCSD level), despite that the SiC(O) bond length of the *ortho*-isomer suggests substantial influence of reverse polarization.

However, not only the SiC bonds are essential. It is important to note that the variations in the CO bond lengths among the three parent silaphenolate isomers **1a**–**3a** are only modest, a finding which is not in line with a variation in the extent of reverse polarization among the silaphenolates. e.g., at CCSD level the variation between the three isomers is merely 0.014 Å (1.252–1.266 Å), and in the all-carbon phenolate the CO bond is 1.260 Å long, *i.e.*, in the middle of the bond length range of the silaphenolates. When regarding the alternation in the CC bond lengths of the ring, the alternation is largest in the *meta*-isomer and smallest in the *ortho*-isomer (0.088 and 0.050 Å, respectively). It is notable that the longest CC bonds in the *meta*-isomer are the two CC bonds flanking the CO bond. In the all-carbon phenolate the CC bond length alternation is 0.060 Å, well within the ranges of the silaphenolates, and when judged from the geometries one may conclude that there are close similarities between these species.

With regard to the substituted *ortho*-silaphenolates, they are also planar, like **1a**. When changing the substituent at Si there is a slight SiC bond length reduction with R = *t*-Bu (**1b**), but with R = SiMe_3_ (**1c**) there is an opposite trend toward elongations of these bonds. The CO bond displays very slight elongations both when R = *t*-Bu and SiMe_3_. The changes in CC bond lengths are small, yet, one can note that the CC bond length alternation decreases when going from **1a** to **1b** (from 0.053 to 0.039 Å at B3LYP level). Still, when regarding the three *ortho*-silaphenolates one can observe that the CO bond essentially corresponds to a modestly elongated C=O double bond, whereas the ring CC and SiC bonds flanking the CO bond tend towards single bonds. The remaining four bonds of the ring are intermediate between single and double bonds in length, and the best description of the *ortho*-silaphenolates should be in terms of resonance structure **II** (Scheme 3).

For the *meta*-silaphenolates the change from R = H (**2a**) to R = *t*-Bu (**2b**) leads to no significant bond length changes, neither in the SiC, CC, nor CO bonds. However, the change to R = SiMe_3_ (**2c**) leads to a slight elongation of the SiC bonds, while the CC and CO bonds remain at similar lengths. When compared to the *ortho*-silaphenolates, the three *meta*-silaphenolates often have short CO bonds and elongated CC bonds flanking the CO bonds, whereas the other CC and the SiC bonds of the ring are shortened. One can thus conclude that also the three *meta*-silaphenolates are described to a fair extent by a resonance structure with a silapentadienyl anionic segment and a C=O double bond, similar as the *ortho*-silaphenolates.

Finally, the two substituted *para*-silaphenolates are planar as well, and for these one observes that the SiC bond lengths of **3b** resemble those of **3a**, whereas slight bond elongations are found for **3c**. Yet, all three *para*-silaphenolates still have SiC bonds which resemble those of silabenzene, *i.e.*, they are intermediate between single and double bond lengths. For **3c** the CO bond is somewhat shorter than for **3a** and **3b**, indicating that this species is more influenced by quinoidal (reverse polarized) resonance structures than the others. This falls in line with the longer SiC bonds of **3c**. The CC bond lengths also reflect influence of such a quinoid resonance structure.

We further investigated the geometries of *ortho*-silaphenolates **1a** and **1c** coordinated by THF-solvated potassium ions. In our computations the potassium ion was coordinated by five THF molecules since we earlier observed in a computational study that potassium silenolates, initially coordinated by a larger number of THF molecules, preferred hexacoordination (the silenolate plus five THF molecules) as additional THF solvent molecules drifted away from the first solvation shell [21].

**Figure 2:**
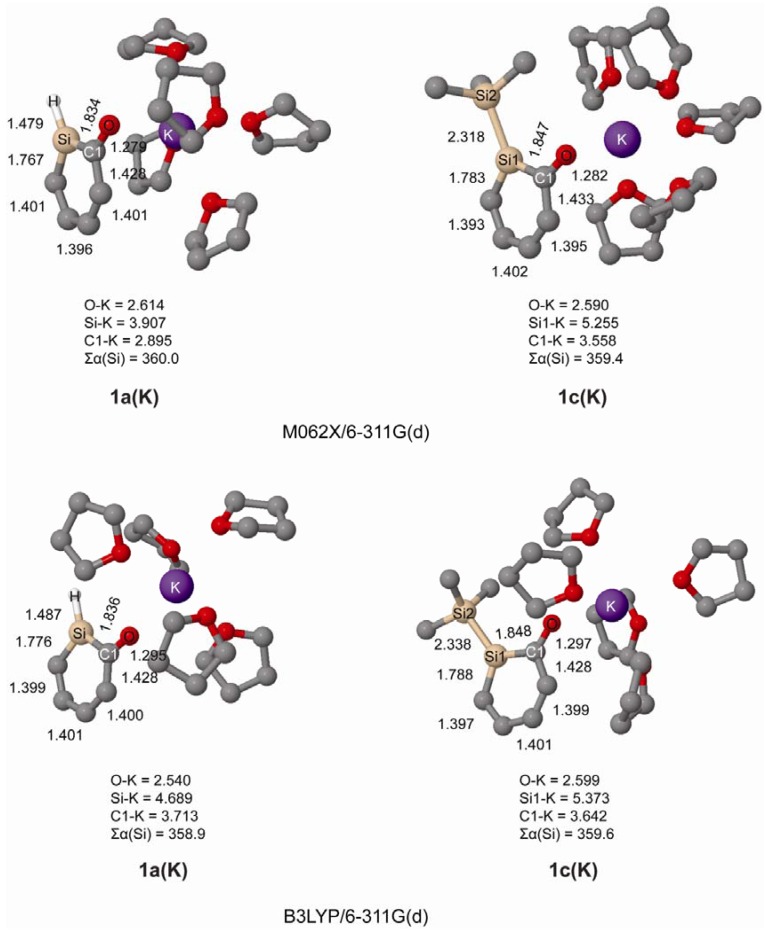
Geometries of K^+^(THF)_5_ solvated silaphenolates **1a(K)** and **1c(K)** calculated at M062X/6-311G(d) (top) and B3LYP/6-311G(d) (bottom) levels of computation. Bond lengths in Å and sum of valence angles (Σα(Si)) in degree. Hydrogen atoms omitted for clarity.

From the computations we find that both species coordinate to K^+^ via their anionic oxygen atoms, and that the K-Si distances are very long as seen in Figure 2. Importantly, at both B3LYP and M062X levels, and for both **1a** and **1c**, one can note significant CO bond elongations upon K^+^(THF)_5_ complexation. One can also observe substantial SiC(O) bond length reductions, while the SiC(H) bonds are only modestly shortened. Thus, the SiC(O) bonds become more intermediate between SiC single and double bonds, even though they still are longer than in the parent silabenzene. With regard to the CC bond length variation it is reduced upon K^+^(THF)_5_ complexation (0.051 *vs.* 0.029 Å in bare *vs.* complexed **1a**, and 0.050 *vs.* 0.031 Å in bare *vs.* complexed **1c** at B3LYP/6-311G(d) level). Clearly, complexation of the potassium ion to both **1a** and **1c** influences the electronic structure of the silaphenolates so that the silaaromatic resonance structure **I** (Scheme 3) with the negative charge placed at the O atom increases in importance. 

### 3.3. Charge Distributions in Silaphenolates

A comparison of the atomic charges calculated with M062X, B3LYP, and MP2 with those from CCSD shows that the two DFT methods give the best agreement with the CCSD results. Whereas the atomic charge at Si calculated by natural population analysis varies extensively among the three parent silaphenolates with **1a** and **1c** having the lowest positive charge, there is a very modest variation in the charge at oxygen (Table 2). For example, at M062X level it varies in the range −0.743 to −0.762 e for **1a**–**1c**, and the charge at O of the all-carbon phenolate is −0.771 e. When considering all nine silaphenolates the variation in the charge at O at M062X level is found within the small range −0.733 to −0.779 e, and similar modest variations are found also at the other computational levels. The charge distributions therefore indicate that the silaphenolates are very similar to the all-carbon phenolates. Indeed, the variation in charge at Si between the three isomers should be related to the positions taken by the Si atom in a pentadienyl fragment for which negative charge localizes at positions 1, 3 and 5.

**Table 2:** Calculated Si and O atomic charges and NICS values of silaphenolates **1a**–**3c**^a^.

Compound	q(Si)				q(O)				NICS(1)_zz_
	M062X	B3LYP	MP2	CCSD	M062X	B3LYP	MP2	CCSD	
**1a**	0.787	0.758	0.838	0.792	−0.743	−0.723	−0.700	−0.726	−18.5
**1b**	1.215	1.190	1.200	-	−0.779	−0.755	−0.729	-	−13.3
**1c**	0.600	0.592	0.636	-	−0.759	−0.725	−0.713	-	−16.5
**2a**	1.131	1.100	1.084	1.139	−0.753	−0.738	−0.710	−0.732	−18.8
**2b**	1.486	1.441	1.415	-	−0.748	−0.736	−0.706	-	−11.8
**2c**	0.926	0.896	0.838	-	−0.751	−0.736	−0.709	-	−16.5
**3a**	0.777	0.782	0.858	0.776	−0.762	−0.744	−0.726	−0.742	−16.6
**3b**	1.158	1.150	1.190	-	−0.770	−0.751	−0.729	-	−13.3
**3c**	0.565	0.584	0.638	-	−0.733	−0.719	−0.697	-	−14.7
**SiC_5_H_6_**	1.168	1.156	1.140	1.158	-	-	-	-	−24.3
**C_6_H_5_O^-^**	-	-	-	-	−0.784	−0.768	−0.745	−0.771	−18.9

^a^ Atomic charges calculated by natural population analysis (NPA) at the four different levels, and nucleus independent chemical shifts (NICS) at GIAO/M062X/6-311+G(d)//M062X/6-311G(d) level.

Further strong indications that the silaphenolates are closely related to the all-carbon phenolate comes from the natural orbital occupancy of the 2pπ(O) natural atomic orbitals (NAOs) in the four species (Table 3). At CCSD level the occupancies of the 2pπ(O) NAOs in **1a**, **1b** and **1c** are 1.56, 1.55 and 1.55 e, respectively, and in the all-carbon phenolate it is 1.60 e. Clearly, the O atom does not participate significantly different in the p-conjugation in the *ortho*-, *meta*-, *para*-silaphenolates and the all-carbon phenolate. Instead, the differences in charge at Si are related to variations in the charge distribution within the silapentadienyl anionic segment, rather than to differences in the extent of reverse polarization exerted by the O atom.

**Table 3:** The natural atomic orbital occupancy of the 2pπ atomic orbital of the oxygen atom of silaphenolates **1a–3a** and the parent all-carbon phenolate ^a^.

Compound	Natural atomic orbital occupancy [2pπ(O)]			
	M062X	B3LYP	MP2	CCSD
**1a**	1.58	1.57	1.55	1.56
**2a**	1.58	1.57	1.54	1.55
**3a**	1.58	1.57	1.56	1.55
**C_6_H_5_O^-^**	1.61	1.60	1.58	1.60

^a^ From calculations using the 6-311G(d) valence triple-zeta basis set.

As can be expected for the substituted silaphenolates, the *t*-Bu group increases the positive charge at Si while the SiMe_3_ group reduces it. Silaphenolate **3c** has the least positive Si atom, closely followed by **1c**. For this reason they should be less prone to dimerize, and are good targets for synthesis. 

### 3.4. Nucleus Independent Chemical Shifts of Silaphenolates

The nucleus independent chemical shift (NICS) index is nowadays one of the most commonly used computational tools for estimation of the aromaticity of a (mono)cyclic compound [32,33]. Several different refinements of the NICS index have been developed since the introduction of the index in 1996 by Schleyer and co-workers. The most sophisticated NICS(0)_πzz_ version, based on only the contributions of the zz (perpendicular) tensor components of the π-MO’s, gives the best results, although the quality of the more readily available NICS(1)_zz_ data (1 Å above the ring center) is also very high [34]. Herein, we have used the latter method.

The NICS(1)_zz_ values of benzene and the parent silabenzene are −30.2 and −24.3 ppm, respectively. We use the latter value as a benchmark that represents the maximal degree of aromaticity that a silaphenolate may display, *i.e.*, an indicator on the importance of resonance structure **I**, Scheme 3. As seen in Table 2 all uncomplexed silaphenolates have NICS(1)_zz_ values that suggest significant influence of aromaticity. Yet, they are less aromatic than the parent silabenzene, in line with an influence of resonance structures described by exocyclic C=O double bonds and silapentadienyl anionic segments (Scheme 3). Further support for this conclusion comes from the NICS(1)_zz_ value of the all-carbon phenolate (−18.9 ppm) which is very close to those of the three parent silaphenolates (−18.5 (**1a**), −18.8 (**2a**), and −16.6 ppm (**3a**), respectively).

However, the substituents at Si also influence the degree of aromaticity. Regardless of isomer, the silaphenolates with a *t*-Bu substituent at Si (**1b**–**3b**) are less aromatic than the corresponding isomers with R = H and R = SiMe_3_ substitution (**1a**–**3a** and **1c**–**3c**, respectively). For the *ortho*- and *para*-isomers, a SiMe_3_ substituent at Si also leads to a slightly reduced aromaticity.

### 3.5. ^13^C and ^29^Si NMR Chemical Shifts of Silaphenolates

As seen in Table 4, the ^29^Si-NMR chemical shifts are influenced by two factors; (*i*) the position of the Si atom in the ring (*ortho*, *meta*, *vs. para*); and (*ii*) the substituent at the Si atom (R = H, *t*-Bu *vs.* SiMe_3_). The δ^29^Si values are calculated to have the most positive values for the *meta*-silaphenolates (**2a**–**2c**), whereas the ^29^Si shifts of the *para*-silaphenolates (**3a**–**3c**) are only slightly more downfield than those of the corresponding *ortho*-silaphenolates (**1a**–**1c**). There is also a clear dependence of the ^29^Si shifts on the substituent R at silicon, with the electron withdrawing *t*-Bu substituent (**1b**–**3b**) giving the most downfield shifted δ^29^Si values while R = H (**1a**–**3a**) giving the least. With regard to the ^13^C-NMR chemical shifts the carbon atom bonded to the oxygen atom is calculated to values in the range 196–242 ppm whereas the other C atoms have resonances in the range 89–165 ppm. The ^13^C-NMR chemical shifts of the C atoms next to the Si atom display some dependence on the substituent, however, the shift variation is smaller for the other C atoms in the ring.

**Table 4:** The ^13^C and ^29^Si-NMR chemical shifts of silaphenolates **1a**–**3c**^a^.

Silaphenolate	Si1	C2	C3	C4	C5	C6
**1a**	64.0	229.7	151.9	138.7	132.7	155.1
**2a**	91.5	123.9	208.4	141.3	160.5	101.7
**3a**	2.0	154.4	160.3	198.2	160.3	154.4
**1b**	126.9	224.5	150.9	136.9	129.7	136.7
**2b**	147.1	109.7	209.5	138.1	159.9	88.8
**3b**	73.2	141.4	157.5	196.2	155.6	141.4
**1c**	81.7	242.4	150.2	142.4	134.1	165.5
**2c**	111.5	134.9	207.1	143.8	161.4	110.7
**3c**	17.3	163.8	161.4	200.7	160.7	165.7

^a^ Chemical shifts calculated at GIAO/M062X/6-311+G(d)//M062X/6-311G(d) level.

### 3.6. Dimerization Aptitudes of Silaphenolates

We also regarded dimerization of the bare (uncomplexed) silaphenolates, as well as silaphenolates complexed by K^+^(THF)_5_. However, it should be noted that far from all dimers were examined, and therefore, this part of the study mainly provides trends. We studied the dimers of **1a**–**3a** and **1c**–**3c**, where the latter species were particularly included as it was found in earlier studies of silenes that silyl groups at Si increase the stability by reducing the partial positive charge at Si [15].

Several different types of dimers can form. First, there are regular [4+2] and [2+2] cycloadducts that exclusively involve the silabenzene rings and in which the sp^2^ hybridized Si atoms of the silaphenolates have been transformed into sp^3^ hybridized Si atoms. In addition, for silaphenolates there exist dimers in which the anionic oxygen atoms bind to the silicon atoms forming strong SiO bonds, and these latter dimer types seem to be of lowest relative energies. Furthermore, the dimers can have head-to-head (Si atoms on same side in the new cycle formed) or head-to-tail (Si atoms placed diagonally in the new cycle) configurations. Finally, the dimers of the *ortho*- and *meta*-silaphenolates can exist as either *endo*- or *exo*-isomers. As noted above, the relative bond strengths in the dimers influence their relative stabilities, but variations in Coulomb repulsion are also important.

**Figure 3:**
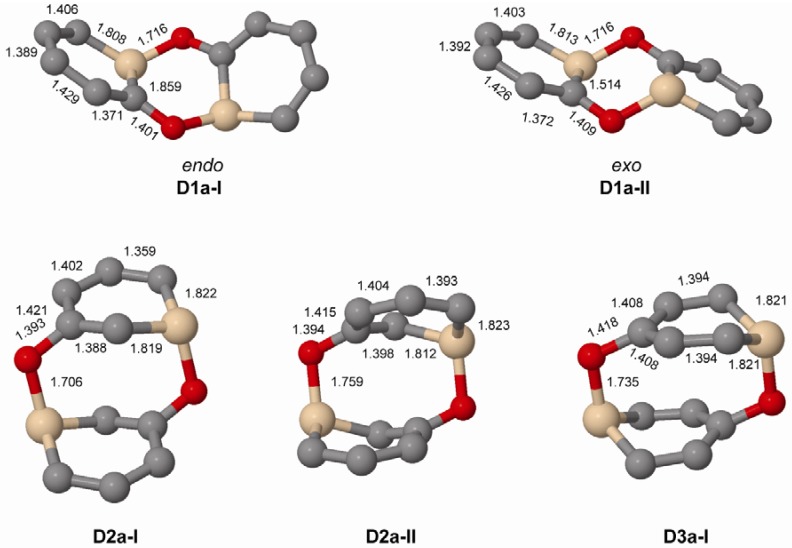
Geometries of the Si-O bonded dimers of **1a**–**3a** (C_2_ symmetry) calculated at M062X/6-31G(d) level. Hydrogen atoms omitted for clarity. Bond lengths given in Å.

**Figure 4:**
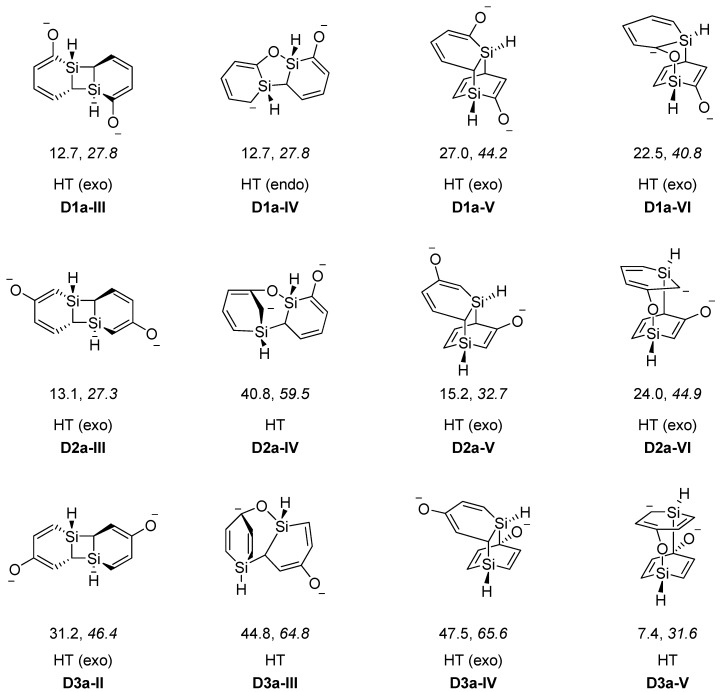
Low-energy [2+2], [4+2] and other dimers of silaphenolates (**1a–3a**) calculated at the M062X/6-311G(d)//M062X/6-31G(d) (normal) and B3LYP/6-31G(d) (italics) levels. Dimerization energies in kcal/mol. From left to right the dimers are; [2+2], [2+3], [4+2], and [4+3] cycloadducts, where the [2+2] and [4+2] cycloadducts involve only Si-C bond formation whereas the [2+3] and [4+3] cycloadducts involve also Si–O bond formation.

When regarding dimers of the unsubstituted silaphenolates (Figure 3 and Figure 4, and Table 5) one can note that for **1a** the dimers with two Si-O bonds (**D1a-I** and **D1a-II**) are more stable than two separate monomers at M062X level, but less stable at B3LYP level. As M062X is an improved functional which is suitable to describe dispersive intramolecular interactions [26], the dimer energies from this method is likely of better quality than those of B3LYP. Other dimers of **1a** (**D1a-III**–**D1a-VI**, Figure 4) are, however, less stable than two monomers, both at M062X and B3LYP levels. Besides the strong Si-O bonds, the **D1a-I** and **D1a-II** dimers have their two pentadienyl anionic segments as distant from each other as possible, whereas the corresponding distances are smaller in **D1a-III**–**D1a-VI**.

The increased impact of the intramolecular Coulomb repulsion also becomes apparent through a comparison of the dimers of **1a** with those of **2a** and **3a** as the repulsion in the latter species is so extensive that they are more stable as monomers. The dimer in which Si-O bonds are formed (**D2a-I** and **D2a-II**, Figure 3) are ~10 kcal/mol less stable than two separate monomers at M062X level, and the [4+2], [2+2] cycloadducts and other dimer types (**D2a-III**–**D2a-VI**) are 13–41 kcal/mol less stable (Figure 4). A similar situation applies to the **D3a** dimers (Table 5, Figure 3 and Figure 4). At B3LYP level, two monomers are more stable than the dimers by 26 kcal/mol and upwards. This should be compared with computational results for the 1-Tbt-silabenzene synthesized by Tokitoh and co-workers [5]. They found this silabenzene to be 10.5 kcal/mol more stable as two monomers than as dimer at B3LYP/6-31G(d) level. From a comparison with the dimerization of this species one can conclude that even the parent *meta*- and *para*-silaphenolates should be stable species. 

When comparing the geometries of the parent silaphenolate dimers (Figure 3) one finds that in the **D2a-II** and **D3a** dimers, the Si-O bonds are slightly longer than in **D1a-I**, **D1a-II** and **D2a-II**. The C-O bonds are also somewhat longer in **D3a** than in the other dimers. Both of these structural features should reflect generally larger intramolecular Coulomb repulsion in the dianionic **D2a-II** and **D3a**.

**Figure 5:**
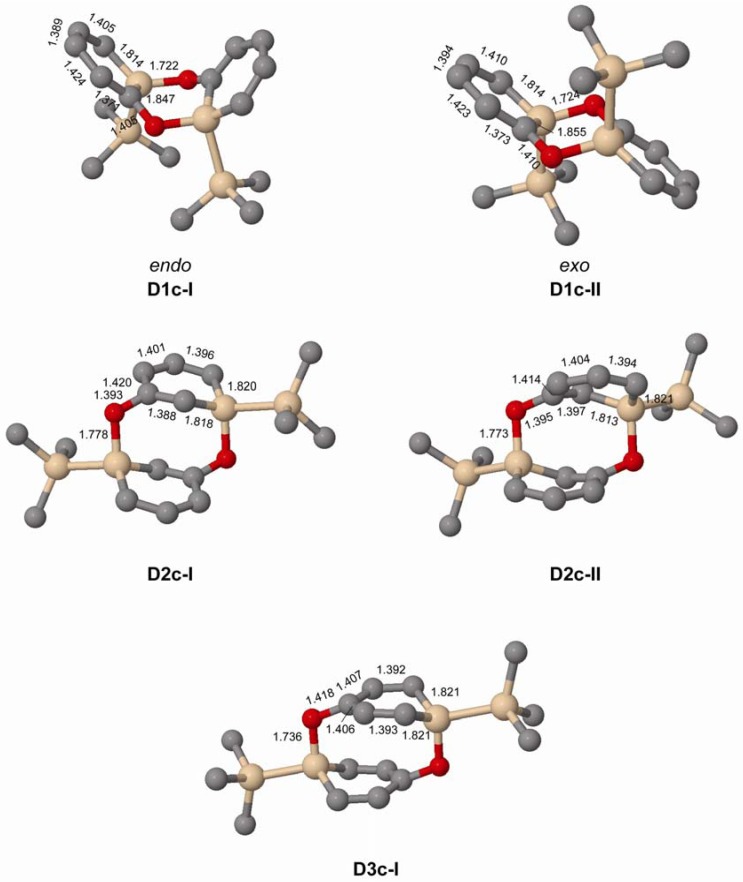
Geometries of doubly Si-O bonded dimers of **1c**–**3c** (C_2_ symmetric) calculated at the M062X/6-31G(d) level. Distances in Å. Hydrogen atoms omitted for clarity.

When changing the Si substituent of the *ortho*-silaphenolate from R = H to R = SiMe_3_ the *endo*-dimer **D1c-I** is slightly destabilized when compared to **D1a-I** whereas the *exo*-dimer **D1c-II** is not destabilized over **D1a-II** (Table 5). This finding can be rationalized by the steric congestion between the two SiMe_3_ groups that occurs in **D1c-I** but not in **D1c-II** (Figure 5). Clearly, more substantial steric bulk than provided by a SiMe_3_ group needs to be exercised by the substituent at Si in order to destabilize the **D1c-II** dimer to the extent that it becomes less stable than two monomers.

**Table 5:** Reaction energies (kcal/mol) for formation of dimers of silaphenolates calculated at the M062X/6-311G(d)//M062X/6-31G(d) (normal) and B3LYP/6-31G(d) (italics) levels ^a^.

Dimers of 1a–3a		Dimers of 1c–3c		Dimers of 1a(K) ^b^		Dimers of 1c(K) ^b^	
Compound	*E_dim_*	Compound	*E_dim_*	Compound	*E_dim_*	Compound	*E_dim_*
**D1a-I**	−9.1, *8.0*	**D1c-I**	−4.1, *16.4*	**D1a(K)-I**	−8.3	**D1c(K)-I**	1.3
**D1a-II**	−10.9, *7.0*	**D1c-II**	−11.7, *13.9*	**D1a(K)-II**	−16.5	**D1c(K)-II**	0.6
**D2a-I**	8.7, *29.7*	**D2c-I**	3.3, *26.9*				
**D2a-II**	10.5, *31.3*	**D2c-II**	6.3, *29.0*				
**D3a-I**	7.4, *31.6*	**D3c-I**	13.3, *39.3*				

^a^ For optimized geometries see Figure 3; ^b^ Here **(K)** symbolizes coordination of a K^+^(THF)_5_ moiety to a silaphenolate monomer.

**Figure 6:**
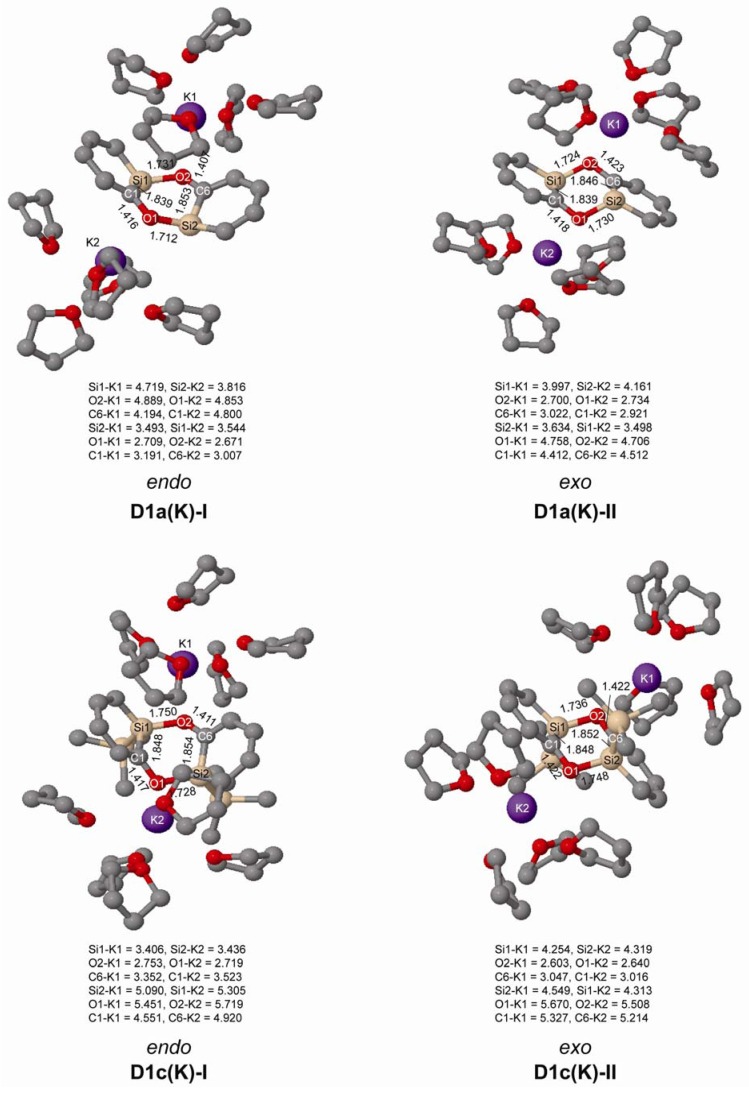
Geometries of the doubly Si-O bonded of **1a(K)** and **1c(K)** dimers solvated by K^+^(THF)_5_ calculated at M062X/6-31G(d) level. Distances in Å. Hydrogen atoms omitted for clarity.

In experiments the silaphenolate will be complexed by a solvated counterion, and through computations of K^+^(THF)_5_ complexed silaphenolate dimer of **1a** and **1c** we find that this also affects the dimerization aptitude (Table 5 and Figure 6). The dimerization energy of **D1a(K)-II** is larger than that of **D1a-II**, yet, for both of the dimers of **1c** with Si–O bonds (**D1c(K)-I** and **D1c(K)-II**) one notes smaller dimerization energies than for the corresponding uncomplexed silaphenolates. For the particular solvent configurations studied here the two **D1c(K)** dimers are slightly less stable than two monomers. However, a very large number of other solvent configurations exist, and it is not unlikely that a few of these will lead to lower energies than the ones discussed herein. 

### 3.7. Silaphenylthiolates

**Scheme 4:**
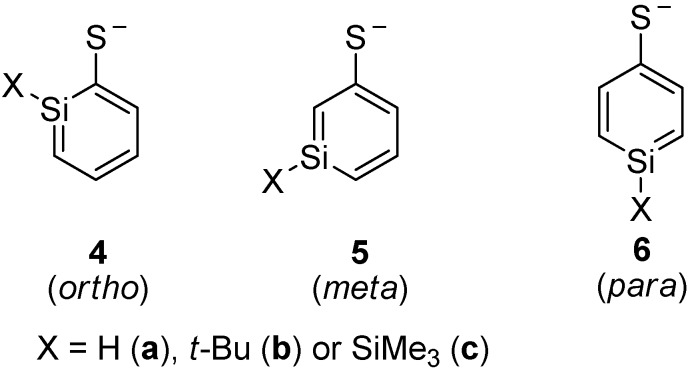
Isomers of silaphenylthiolates.

Silaphenylthiolates **4a**–**6c**, which are analogous to silaphenylthiolates **1a**–**3c**, were examined as well (Scheme 4). With regard to the parent silaphenylthiolates **4a**–**6a**, the *ortho*- and *meta*-isomers are isoenergetic, whereas the *para*-isomer is of higher energy (Table 6). For these species one can note that the method variation is smaller than for the silaphenolates. With a SiMe_3_ or *t*-Bu substituent at Si, the *ortho*-isomer is the most stable isomer whereas the *meta*-isomers are the second most and the *para*-isomers the least stable ones. 

**Table 6:** Calculated relative energies (kcal/mol) of silaphenylthiolates **4a**–**6c**^a^.

Compound	*E*_rel_				*H* ^298^	D*G*^298^
	M062X	B3LYP	MP2	CCSD	M062X	M062X
**4a**	0.0, *0.0*	0.0	0.0	0.0	0.0	0.0
**4b**	0.0, *0.0*	0.0	0.0	-	0.0	0.0
**4c**	0.0, *0.0*	0.0	0.0	-	0.0	0.0
**5a**	0.3, *0.1*	0.6	0.8	-0.4	0.3	0.5
**5b**	3.1, *3.1*	2.1	4.1	-	2.8	4.0
**5c**	4.8, *4.6*	3.4	5.1	-	3.9	4.7
**6a**	3.7, *3.7*	3.5	3.4	3.2	4.0	4.2
**6b**	8.0, *8.2*	6.3	7.6	-	7.3	7.8
**6c**	5.7, *6.0*	3.5	5.3	-	5.6	2.6

^a^ Values in normal print obtained from geometry optimizations at the corresponding levels with the 6-311G(d) basis set, and values in italics obtained from single-point energy calculations using the 6-311+G(d) basis set at the corresponding level.

**Figure 7:**
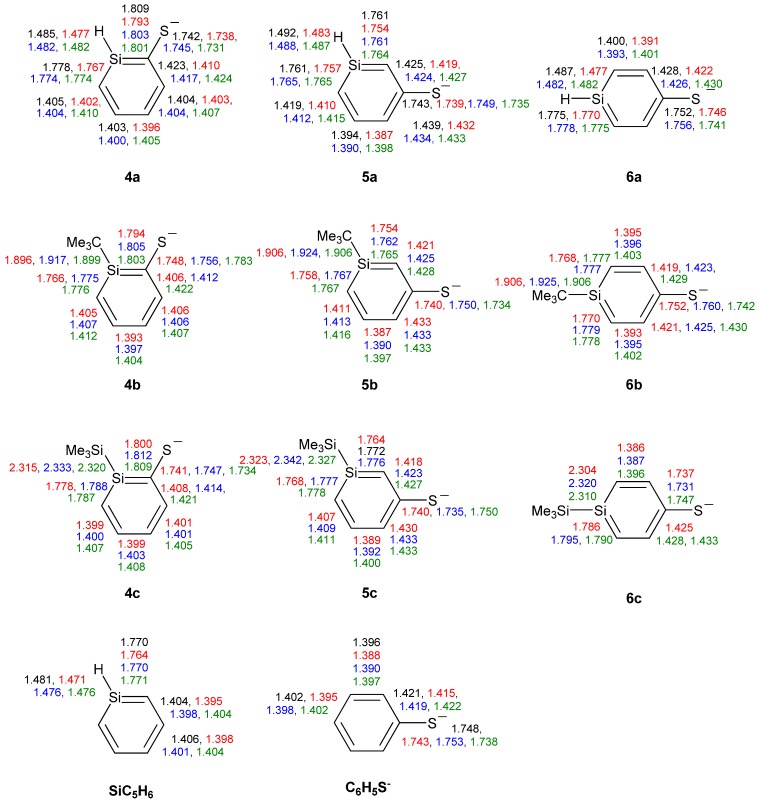
Bond lengths (Å) of silaphenylthiolates **4a**–**6c**, parent silabenzene, and phenylthiolate C_6_H_5_S^-^ calculated at CCSD/6-311G(d) (black), M062X/6-311G(d) (red), B3LYP/6-311G(d) (blue), and MP2/6-311G(d) (green) levels. All sums of valence angles at Si (Σα(Si)) equal 360.0°.

Among the three *ortho*-silaphenolthiolates **4a**–**4c** one sees much less SiC(S) bond elongation than SiC(O) bond elongations among the corresponding **1a**–**1c** (Figure 7). The other bond lengths of the rings are very similar to those of the parent silabenzene. With regard to the *meta*- and *para*-silaphenylthiolates one can observe similar bond length variations as in the corresponding silaphenolates, yet, the variations are significantly smaller. Also, the CS bond lengths of **4a**–**6c** show a very small variation and resemble that of the parent phenylthiolate. Indeed, all phenylthiolates display CS bong lengths which are close to those of regular C(sp^2^)-S and C(Ar)-S single bonds as typical C(sp^3^)-S, C(sp^2^)-S, and C(Ar)-S single bond lengths are 1.817, 1.751, and 1.773 Å, respectively, while the normal C(sp^2^)=S double bond length is 1.599 Å [35]. This suggests that silaromatic resonance structures of type **I** (Scheme 3) contribute much to the electronic structures of silaphenylthiolates. 

When compared to the silaphenolates, the silaphenylthiolates have a less polarized charge distribution (Table 7). In general, the charge at S is less negative and the charge at Si is less positive than at the O and Si atoms in the corresponding silaphenolates. However, this lowered negative charge at S is predominantly due to differences between O and S in the strengths of inductive electron withdrawal. The natural atomic orbital occupancies of the 3pπ(S) NAOs in silaphenylthiolates **4a**, **5a**, and **6a** are higher than in the 2pπ(O) NAOs of the analogous silaphenolates (Table 3 and Table 8). This finding again thus suggests larger contribution of silaaromatic resonance structures of type **I** (Scheme 3) in the silaphenylthiolates than in the silaphenolates. This observation also agrees with the NICS(1)_zz_ values which on average are a little bit more negative in **4a**–**6c** than in **1a**–**3c** (Table 2 and Table 7).

**Table 7:** Calculated Si and S atomic charges (e) and NICS values (ppm) of silaphenolates **4a**–**6c**^a^.

Compound	q(Si)				q(S)				NICS(1)_zz_
	M062X	B3LYP	MP2	CCSD	M062X	B3LYP	MP2	CCSD	
**4a**	1.092	1.072	1.078	1.095	−0.490	−0.475	−0.480	−0.498	−18.0
**4b**	1.467	1.433	1.409	-	−0.514	−0.501	−0.494	-	−15.5
**4c**	0.881	0.860	0.845	-	−0.495	−0.476	−0.478	-	−18.0
**5a**	1.123	1.103	1.078	1.127	−0.506	−0.499	−0.500	−0.507	−18.5
**5b**	1.478	1.442	1.403	-	−0.502	−0.498	−0.497	-	−14.0
**5c**	0.909	0.890	0.834	-	−0.505	−0.499	−0.500	-	−17.7
**6a**	0.943	0.926	0.955	0.957	−0.529	−0.514	−0.524	−0.535	−17.3
**6b**	1.314	1.285	1.275	-	−0.545	−0.531	−0.533	-	−15.1
**6c**	0.715	0.706	0.722	-	−0.498	−0.473	−0.482	-	−17.0
**SiC_5_H_6_**	1.168	1.156	1.140	1.158	-	-	-	-	−24.3
**C_6_H_5_S^-^**	-	-	-	-	−0.546	−0.540	−0.542	−0.549	−21.5

^a^ Atomic charges calculated by natural population analysis (NPA) at the four different levels, and nucleus independent chemical shifts (NICS) at GIAO/M062X/6-311+G(d)//M062X/6-311G(d) level.

**Table 8:** The natural atomic orbital occupancy of the 3pπ atomic orbital of the sulfur atom of silaphenylthiolates **4a**–**6a** and the parent all-carbon phenylthiolate ^a^.

Compound	Natural atomic orbital occupancy [3pπ(S)]			
	M062X	B3LYP	MP2	CCSD
**4a**	1.73	1.71	1.70	1.72
**5a**	1.72	1.71	1.69	1.71
**6a**	1.74	1.72	1.72	1.73
**C_6_H_5_S^-^**	1.76	1.74	1.74	1.75

^a^ From calculations using the 6-311G(d) valence triple-zeta basis set.

The silaphenylthiolates may also have a tendency to dimerize, however, at this time we refrained from investigating this process. Since the Si–S bond is weaker than the Si–O bond, and as the silaphenylthiolates overall are slightly more aromatic, one may conclude that they potentially have dimerization aptitudes which resemble those of silabenzenes. It is therefore likely that the substituents at the Si atom need to be bulkier than for the silaphenolates.

## 4. Conclusions

Silaphenolates and silaphenylthiolates are organosilicon species with sp^2^ hybridized silicon atoms and they are all extensively influenced by aromaticity, although not as extensively as the parent silabenzene according to nucleus independent chemical shift (NICS) data. Their dimerization aptitude is clearly hampered by extensive Coulomb repulsion in the dimer; whereas dimerization of the parent *ortho*-silaphenolate is still exothermic dimerization of the parent *meta*- and *para*-silaphenolates are endothermic. Also, the dimerization of *ortho*-silaphenolates can be turned endothermic through moderate bulk at Si exerted by, for example, a SiMe_3_ group.

To conclude, the silaphenolates, and possibly also silaphenylthiolates, should be interesting targets for synthesis as they could constitute novel (small) compound classes influenced by silaaromaticity.
